# Assessing success in transitioning of young adults from pediatric to adult kidney practice

**DOI:** 10.1186/s12882-019-1665-7

**Published:** 2020-01-13

**Authors:** Ben Joslin, Craig Langman, Laura Nishi, Cybele Ghossein

**Affiliations:** 1 Division of Nephrology, Northwestern University Feinberg School of Medicine, Chicago, IL USA; 2 Northwestern University Feinberg School of Medicine and The Ann and Robert H. Lurie Children’s Hospital of Chicago, Chicago, IL USA; 3 Division of Nephrology and Hypertension, Northwestern Medicine, Chicago, IL USA

**Keywords:** Pediatrics, Transition of care, Transition clinic, Adolescent medicine, Chronic kidney disease

## Abstract

**Background:**

Transfer from a pediatric to an adult medical setting is associated with many barriers. Additionally, there are little data on patients’ assessment of the transition process itself. 3 years ago at Lurie Children’s Hospital of Chicago, we established a kidney transition program with the help of an adult nephrologist, physician assistant (PA) and social worker (LCSW). After 18 months, we evaluated the patients’ perception of the program as part of a continuous quality initiative process.

**Methods:**

Patients who had transitioned from pediatric care and were seen at least once in the adult nephrology clinic were anonymized and asked to take an established 5-point Likert scale survey. Survey questions addressed readiness to transition, the transition process itself, and the perception of adult care. Surveys were followed with semi-structured interviews. 3 readers rated each response as either “negative,” “neutral,” or “positive.” Average, standard deviation and reader reliability were calculated. The readers also selected a word that best depicted each response and those most-common words were counted by question and overall.

**Results:**

17 out of 42 patients (40%) completed the survey. Average age at transition (mean + SD) was 20 + 2 years; the majority of patients (82%) felt ready to transfer to adult care but only 59% felt they were consulted on the timing. 88% of patients felt having a transition appointment and meeting the adult care providers in the pediatric setting to be valuable. Although 94% of patients ultimately felt comfortable in the adult care environment, 18% experienced noticeable differences in treatment recommendations. 13 semi-structured interviews were conducted. Overall, the patients responded positively (3 + 0, 100% reader reliability) to the transition. But, when asked what could have improved the transition, the word the patients used most was, “earlier.”

**Conclusion:**

Young adults (YA) transitioning to adult care often feel ready to transition earlier than their transfer of care date. They subjectively benefit from a transition program that outlines the process of transferring their care. Many YA patients would benefit from a transition program that bolsters patient independence during early adult care visits.

## Background

There is a growing body of research on transitioning patients with chronic conditions from pediatric to adult care [1–3]. Young adult patients face many challenges throughout this transition process. Some challenges include: difficulty navigating adult healthcare systems, gaps in insurance coverage, lack of health self-efficacy, and limited experience in self-management [4]. Transition programs/clinics have been developed to support the transfer to adult care and to reduce patient vulnerabilities and interruptions in care. The International Society of Nephrology and the International Pediatric Nephrology Association released a consensus statement outlining the ideal clinical management of young adult patients [5]. Since these guidelines were released in 2011, there has been a growing interest in evaluating transition programs. While most research has focused on clinical outcomes to determine the effectiveness of transition programs, little work has been done to evaluate patients’ viewpoints of a transition program.

In 2014, physicians, a physician assistant (PA), and a licensed clinical social worker (LCSW) at Lurie Children’s Hospital of Chicago (LCH) and Northwestern Memorial Hospital (NMH) established a transition clinic in order to chaperone patients from pediatric to adult nephrology care. Young adult patients followed at LCH were identified by their pediatric nephrologist as ready to transition to adult care and an appointment made in the transition clinic. The timing of their transition was determined by the pediatric nephrologist. Transition began with a discussion between patients, patients’ parents, and pediatrician to outline expectations of the transfer. Patients and their parents were given time to ask questions and provide input.

The monthly transition clinics occurred at LCH, which is connected by a bridge to the adult hospital. An adult nephrologist, PA, and LCSW would meet with the providers from LCH to share medical records and collaborate on the appropriate treatment approach for each patient. Appointments were run primarily by NMH staff. The patients and their parents met with each of the adult care providers to review the needs of the patient and parent. An adult nephrologist reviewed the medical history and plan of care, while the LCSW assessed emotional needs as well as insurance and social support. If patients had a transition plan in another subspecialty, the adult team did not interfere with that transition plan. The PA provided a “readiness to transition” questionnaire as well as all appropriate phone numbers, maps and “how to navigate an adult hospital” information sheet. A discussion of the patient’s roles and responsibilities was undertaken by each caretaker during the appointment. Examples of these responsibilities include scheduling of their own appointments and filling their own prescriptions. Instructional pamphlets were provided to the patients to take home. Referrals were made by the adult provider. The patient graduated to NMH for their care after the transition appointment unless the adult care team or the patient felt that the patient would benefit from another transition clinic meeting.

After 18 months, 42 pediatric patients were transitioned to adult care through the transition clinic. The purpose of this current project was to evaluate the patients’ perceptions of the transition clinic program. While we valued our program, we sought to learn from those participating in it to assess how it could be improved. Our objectives were to assess patient’s perceptions of the transition in three time points: preparing for transition, the transition process itself, and adjusting to adult care.

## Methods

Patients who had transitioned from pediatric care and were seen at least once in the adult clinic at the study hospital were asked to take an established 5-point Likert scale survey six to twelve months after transitioning. Patients who had received a kidney transplant were not included in this study because they participated in a different transition program. The survey questions were modified from a survey used by Jensen et al. (2015) to evaluate a rheumatology transition program [6]. The survey was administered through the online platform, Qualtrics®. Patients completed the survey either on an iPad in the clinic or on their own device. Survey questions addressed preparing for the transition, the transition process itself, and adjusting to adult care (Table 3 in Appendix 1). Responses were categorized into Top 2 Box (“strongly agree” or “agree”), neutral, and Bottom 2 Box (“strongly disagree” or “disagree”). All responses were anonymized and analyzed by a research coordinator not involved in the transition program. The responses were uploaded to STATA (StataCorp. 2015. Stata Statistical Software: Release 14. College Station, TX: StataCorp LP.) to collect the mean, standard deviation, and variance for each question.

Two to five days after completing the survey, patients were contacted to schedule semi-structured interviews conducted over the phone by the research coordinator. Interview questions were sampled from an interview used by Reid et al. (2004) to evaluate the transition of cardiology patients and modified by our team members (Table 4 in Appendix 1) [7]. Patient responses were transcribed into Microsoft Word by the research coordinator. Three readers rated each response on a 3-point Likert scale as either “negative (1),” “neutral (2),” or “positive (3).” Average, standard deviation and reader reliability were calculated using STATA. Additionally, and independently, the readers also selected a word that best depicted each response and those most-common words were counted by question and overall.

## Results

42 patients completed one adult visit after the transition clinic program. Median age of transition was 20 years old + 2 years. The majority of transition patients were male and not Hispanic or Latino. Ethnicity was derived from the patients’ charts and it should be noted that 33% declined to state their ethnicity. There was a diverse array of primary diagnoses but the majority of patients were treated for glomerular disease or hypertension (Table 1). Patients who had received a transplant did not participate in this study.

**Table 1:** Baseline characteristics of patients who participated in the transition program, survey and interviews

	Participants in transition program (*n* = 42)	Survey respondents (*n* = 17)	Interview respondents (*n* = 13)
Age at Transition (median, range)	20 (18–25)	20 (17–23)	20 (18–22)
Male (n, %)	25 (60%)	9 (53%)	6 (46%)
*Ethnicity (n, %)*			
Not Hispanic or Latino	18 (43%)	12 (71%)	10 (77%)
Hispanic or Latino	10 (24%)	4 (24%)	2 (15%)
Declined	14 (33%)	1 (5%)	1 (8%)
*Disease category (n, %)*			
Glomerular Disease	25 (60%)	9 (53%)	7 (54%)
Tubular Interstitial Disease	4 (9%)	0	0
Hypertensive/ Vascular Disease	5 (12%)	3 (18%)	2 (15%)
Other	8 (19%)	5 (29%)	4 (31%)
*Disease Severity (n,%)*			
	CKD Stages 1–2	8 (48%)	5 (38%)
	CKD Stages 3–5	9 (52%)	8 (62%)

### Surveys

**Fig. 1:**
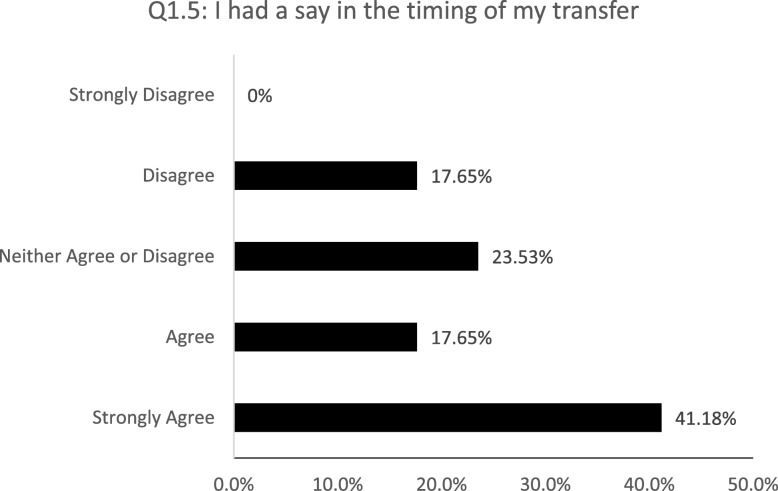
Patients’ perception of the timing of the transfer. 5-point Likert scale: strongly disagree (1), disagree (2), neither agree or disagree (3), agree (4), and strongly agree (5)

17 out of the 42 patients completed the survey. Median age of respondents was 20 years old, male (53%), and transitioned with a diagnosis of CKD stages 3–5 (52%) (Table 1). The majority of patients felt their parents were ready for them to transfer to adult care (mean + SD; 4.35 + 0.84) and that the timing of the transfer was appropriate (4.47 + 0.85). However, while 82% of patients felt ready to transfer to adult care (4.41 + 0.77), only 59% strongly agreed or agreed that they were consulted on the timing (3.82 + 1.15) (Fig. 1).

**Fig. 2:**
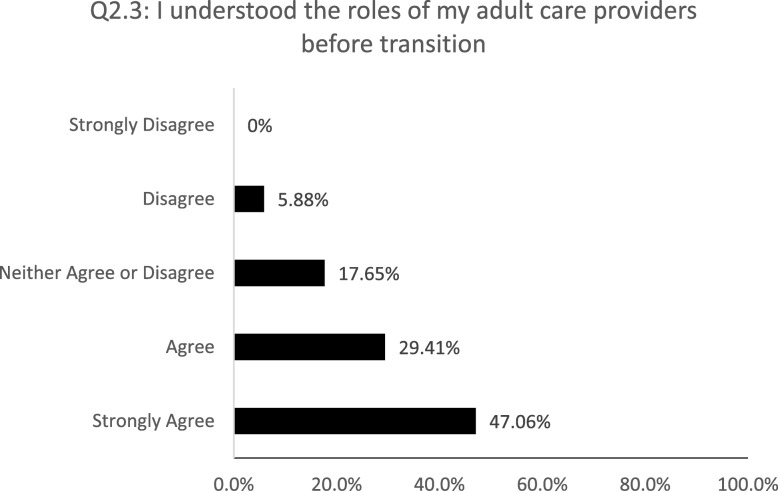
Patients’ understanding of the roles of their adult care providers before their transition. 5-point Likert scale: strongly disagree (1), disagree (2), neither agree or disagree (3), agree (4), and strongly agree (5)

All patients believed that they received enough information about the transition to an adult setting (100%, 4.53 + 0.5). Most patients (88%, 4.59 + 0.69) believed there was good collaboration between pediatric and adult care. 88% of patients felt having a transition appointment and meeting the adult care providers in the pediatric setting to be valuable (4.47 + 0.7), although 24% did not understand the roles of their adult care providers (Fig. 2).

Ultimately, 94% of patients felt comfortable in the adult care environment (4.65 + 0.59) while only 29% reported they were struggling with their care (2.53 + 1.38). 88% of patients stated they were happy with the care (4.47 + 0.85) and felt taken care of in the adult care setting (4.59 + 0.84). While 94% of patients felt that their adult care provider was well informed about their condition (4.71 + 0.75), 24% did not know what was expected of themselves as patients and of their providers.

Many patients (76%, 3.88 + 1.02) felt their treatment recommendations in adult care were similar to those received during their pediatric care. All patients understood how their medications related to their adult treatment plan (100%, 4.82 + 0.38) and most patients felt that they could manage their medications well on their own (82%, 4.12 + 0.83). However, only 53% were strongly confident about navigating their first adult care appointment on their own (76%, 4.29 + 0.82).

### Semi-structured interviews

13 semi-structured interviews were conducted from the 17 patients who completed the survey. Median age of respondents was 20 years old, female (54%), and transitioned with a diagnosis of CKD stages 3–5 (62%) (Table 1). The reader ratings and common word identified in each response can be found in Table 2.

**Table 2:** Reader reliability and reader common word identification of semi-structured interview responses. Readers rated responses as negative (1), neutral (2), or positive (3)

No.	Question	Mean	Std. Dev	Reader Reliability	Reader Common Word	Common Word Count
1	What were the major differences you noticed between pediatric and adult care?	2.3	0.35	87%	Independent	4
2	What were the major similarities you noticed between pediatric and adult care?	3	0	100%	Professional	5
3	What’s the staff like at Lurie/NM?	2.7	0.08	97%	Professional	4
4	What was your transition appointment at Lurie like?	2.4	0.27	90%	Comfortable	5
5	How Involved were your parents/caretakers?	2.7	0.32	90%	Independent	17
6	When asked questions about your care, how’d you feel?	2.8	0.27	91%	Comfortable	9
7	How was the timing of this appointment?	2.4	0.19	93%	Appropriate	8
8	How was your waiting room experience at Lurie?	2.3	0.12	95%	Comfortable	6
9	How was the environment at Northwestern Memorial Hospital?	2.5	0.24	91%	Overwhelming	8
10	How did you feel when asked questions at Northwestern Memorial?	2.6	0.16	94%	Comfortable	11
11	What was your experience in the waiting room?	2.5	0.2	92%	Isolated	4
12	Did you experience anything unexpected?	2.6	0.27	91%	Independent	7
13	What else could we have done to make your transition from from pediatric care to adult care a good experience?	2.5	0.35	87%	Earlier	5

Three raters agreed that the patients experienced positive similarities between pediatric and adult care (3.0 + 0.00, 100%). The most common word in patients’ responses from this question was “professional” (*n* = 5). For example, “The doctors are very professional in both hospitals” (Interview 7.2). Alternatively, most patients were unaffected by the differences between pediatric and adult care (2.3 + 0.35, 87%).

Overall, the patients were not concerned with the transition appointment itself (2.4 + 0.27, 90%) and the timing of the appointment (2.4 + 0.19, 93%). The most common word patients used in their response to the timing of the appointment was, “appropriate” (*n* = 8). Most felt positively about being asked questions during the transition appointment (2.8 + 0.27, 91%). Not only were the participants engaged during the transition appointment, most felt their parents were also involved (2.7 + 0.32, 90%) in the discussion.

Patients had a more positive waiting room experience in Northwestern Memorial Hospital (2.5 + 0.20, 92%) than in Lurie Children’s Hospital (2.3 + 0.12, 95%). The most common words used by patients to describe the waiting rooms were “isolated” (*n* = 4) and “comfortable” (*n* = 6), respectively. Patients seemed to prefer the environment at Northwestern (2.5 + 0.24, 91%) and felt comfortable answering questions there (2.6 + 0.16, 94%).

The majority of patients did not experience anything unexpected during or after the transition (2.6 + 0.27, 91%). “Independence” (*n* = 7) was the word that was used most in their responses to this question. When asked what could be done to improve their transition, the word the patients used most was “earlier” (*n* = 5).

## Discussion

As the care of pediatric chronic medical conditions improves, the number of young adults (YA) with chronic illness transitioning into adult care is increasing [8]. Transitioning care is a complex process. Patients are transitioning their medical care team during a time that is filled with social and emotional challenges. The consequences of poor transitional care have been well established with YA's having an increase rate of poor medical and socioeconomic outcomes [9]. Many health care organizations have recognized the importance of and advocated for improved pediatric to adult transitional care [10].

While much has been written about the readiness to transition, less has been published about the process once the young adult has had a chance to experience an adult medical setting. In adult as compared to pediatric medical settings, increased patient input is required to set and attend appointments, communicate with providers, fill prescriptions, when seeking referrals, as well as other challenges. Our group sought to evaluate the experience of YA patients who had participated in our nephrology transition program and had at least one appointment in the adult care setting at our hospital through surveys and semi-structured interviews.

While only 40% of transition patients participated in the survey, respondents generally represented the transition population in age of transition (20 years) and sex (male). The majority of interview respondents (62%) transitioned with a diagnosis of CKD 3–5 suggesting that results may be representative of a population with greater disease severity. Overall, YA patients had positive perceptions about the transition program as it helped them feel comfortable in the adult care environment. We were, however, able to identify components of our program that could benefit from some improvement.

YA patients who transition to adult renal care should have input on the timing of their transition. The timing of transfer to adult care has been identified as a challenging component of the transition process because physicians must time transition of each patient individually [11]. It is important to distinguish between the patient’s readiness and that of the physician and parents. Uniformly, our patients felt they had little input on the timing of the transition. This is not inconsistent with previous published studies showing that patients are often not included in either the decision to transition or the timing [12, 13]. Not including patients in the timing of transition decision may contradict the notion of the YA evolving development of self-identity and independence. While this may not have affected the success of the transition process, it was a shared experience. Consideration of the patient’s input regarding transition timing should be included as an important component of a transition program.

Semi-structured interview responses further demonstrate that patients transitioning to adult care prefer to transition earlier. When asked what could have been done to improve the transition, the word patients used most frequently was “earlier.” Our interview results found that many patients yearn to transition to adult care earlier because they feel out of place in younger children-focused pediatric waiting rooms and care settings. Interview responses suggest that they prefer the “isolated” adult waiting rooms that are calmer than the pediatric waiting rooms full of younger children. Acknowledging the psychosocial experiences of adolescences and young adults in a pediatric environment, may lead to consideration of earlier transition.

Research indicates that successful transition to adult care requires a structured, individualized transition clinic where pediatric and adult providers collaborate on a care plan [14]. Despite this, many chronically ill YA's do not participate in established transition programs and/or are not educated about their transition process [15]. In our survey, all of our patients felt they had enough information about transitioning to the adult care setting. Additionally, 88% of patients felt having a transition appointment and meeting the adult care providers in the pediatric setting was valuable. These results align with previous research on the positive influence of pediatric and adult care providers collaborating in the transition [16].

A major difference between pediatric and adult care centers is multidisciplinary team support, usually more plenteous in pediatrics and limited in adult settings [4]. In our adult care setting, patients are seen by a physician assistant rather than nurse practitioner as in the pediatric setting. During the transition clinic appointment, patients are introduced to all members of the adult care team. Despite this, 24% of patients indicated that they did not understand each of the providers’ roles. Interview responses indicated that some patients did not understand the roles of the physician assistant. Furthermore, interview responses suggested that some patients were confused about being referred to see multiple physicians in the adult care setting. Thus, patients transitioning to adult care would benefit from a more comprehensive description of various care providers and referrals in the adult care environment.

Ultimately, patients who were part of the transition program felt comfortable in the adult care setting. The survey results demonstrate that patients were happy in the adult environment, felt taken care of by staff, and that their adult providers were well informed about their condition and treatment options. The patients felt their adult care providers were “professional” and “empathic,” respectively. Establishing trust between YA and health care worker can often be challenging. The strength of the collaboration between pediatric and adult providers contributes to establishing a professional and caring atmosphere and ultimately trust for YA patients.

Some YA patients would benefit from a transition program that bolsters patient independence during early adult care visits. While patients yearn to transition for independence, some patients face early challenges navigating new responsibilities in their adult care setting [17]. When asked about anything unexpected in their adult care setting, most patients were surprised about their new sense of “independence.” Only 53% of patients reported being strongly confident in navigating the initial adult care appointment on their own. These results demonstrate that young patients initially struggle with their new sense of independence in adult care settings and would benefit from activities and tools that help them manage their new independence.

## Conclusions

Patients’ perceptions of their transition to adult care is instrumental to improving the quality of transition clinic programs. Our results suggest that transition programs will benefit from communicating with patients regarding their perception of transition timing. The pediatric team at LCH now discusses the opportunity to transition to adult care earlier with most patients. Similarly, conversations about the roles of all adult care providers and how to navigate between them should be clear. The adult care providers at NMH now incorporate a discussion about the roles of providers (e.g. PA, RN, and MD) and acknowledge that treatment may change with moving to the adult hospital. Transition programs should also be expanded with additional activities that nurture patients’ independence during early adult care visits. Additional coordination with other specialty transition programs is another planned improvement to our transition program. While these modifications may improve patients’ opinions of the transition program, future work is needed to determine whether they will improve the clinical outcomes based on these patients’ transition experiences. Our findings are limited by the majority of interview respondents transitioning with more severe kidney disease and not taking into account patients who transitioned from outside of our clinic. Nonetheless, we maintain that patients’ perceptions of their transition timing should be considered by physicians, especially in the severely ill.

## Data Availability

The datasets used and/or analyzed during the current study are available from the corresponding author on reasonable request.

## Appendix 1

**Table 3:** Online survey administered to pediatric patients who were seen at least once in an adult care setting. Survey is broken down into three timepoints; preparing for transition, transition, and adjusting to adult care

No.	Preparing for Transition
1	I was ready to transfer to adult care.
2	My parents were ready to transfer to adult care.
3	The transfer to adult care was announced in a timely manner.
4	The timing of the transfer was just about right.
5	I had a say in the timing of the transfer.
No.	**Transition**
1	There was good collaboration between pediatric and adult care.
2	I received enough information about the transfer to adult care.
3	I understood the roles of my adult care providers before the transition.
4	Meeting the adult care practitioner beforehand was useful.
5	I was well prepared for the transfer to adult care.
No.	**Adjusting to Adult Care**
1	I felt comfortable in the adult care environment.
2	I am happy with the care I receive in the adult care setting.
3	I was taken care of very well in the adult care setting.
4	When I first met my adult care provider, I knew exactly what was expected of me and what I could expect from him/her.
5	My new care provider was well informed about me and my condition.
6	I have confidence in my adult health care providers.
7	The members of the adult care staff were sensitive to my needs.
8	The way of working and dealings with patients in adult care are similar to what I was used to in pediatric care.
9	Treatment recommendations in the adult care setting are similar to those I used to receive in pediatric care.
10	I don’t really experience many differences between pediatric and adult care.
11	I can manage my medical care well on my own.
12	I felt comfortable navigating the first adult care appointment on my own.
13	I’m still struggling with being cared for in the adult environment.
14	I don’t understand how my medications relate to my treatment plan.
15	I understand how my current insurance coverage relates to my treatment plan.

## Appendix 2

**Table 4:** Semi-structured interview conducted over the phone with patients who completed the survey

No.	Interview Question
1	What were the major differences you noticed between pediatric and adult care?
2	What were the major similarities you noticed between pediatric and adult care?
3	What’s the staff like at Lurie/NM?
4	What was your transition appointment at Lurie like?
5	How involved were your parents/caregivers?
6	When asked questions about your care, how’d you feel?
7	How was the timing of this appointment?
8	How was your waiting room experience at Lurie?
9	How was the environment at Northwestern Memorial Hospital?
10	How did you feel when asked questions at Northwestern Memorial?
11	What was your experience in the waiting room?
12	Did you experience anything unexpected?
13	What else could we have done to make your transition from pediatric care to adult care a good experience?
